# Effects of Metal Micro and Nano-Particles on hASCs: An In Vitro Model

**DOI:** 10.3390/nano7080212

**Published:** 2017-08-03

**Authors:** Silvia Palombella, Cristina Pirrone, Federica Rossi, Ilaria Armenia, Mario Cherubino, Luigi Valdatta, Mario Raspanti, Giovanni Bernardini, Rosalba Gornati

**Affiliations:** 1 Department of Biotechnology and Life Sciences, University of Insubria, via J.H. Dunant 3, 21100 Varese, Italy; s.palombella@studenti.uninsubria.it (S.P.); cristina.pirrone@hotmail.it (C.P.); federica.rossi@uninsubria.it (F.R.); i.armenia@uninsubria.it (I.A.); mario.cherubino@uninsubria.it (M.C.); luigi.valdatta@uninsubria.it (L.V.); giovanni.bernardini@uninsubria.it (G.B.); 2 Department of Medicine and Surgery, University of Insubria, Via Guicciardini 9, 21100 Varese, Italy; mario.raspanti@uninsubria.it; 3 The Protein Factory Research Center, Politecnico of Milano, ICRM-CNR Milano and University of Insubria, Via Mancinelli 7, 20131 Milano, Italy

**Keywords:** cytotoxicity, cellular uptake, cell morphology, gene expression, adipose stem cells

## Abstract

As the knowledge about the interferences of nanomaterials on human staminal cells are scarce and contradictory, we undertook a comparative multidisciplinary study based on the size effect of zero-valent iron, cobalt, and nickel microparticles (MPs) and nanoparticles (NPs) using human adipose stem cells (hASCs) as a model, and evaluating cytotoxicity, morphology, cellular uptake, and gene expression. Our results suggested that the medium did not influence the cell sensitivity but, surprisingly, the iron microparticles (FeMPs) resulted in being toxic. These data were supported by modifications in mRNA expression of some genes implicated in the inflammatory response. Microscopic analysis confirmed that NPs, mainly internalized by endocytosis, persist in the vesicles without any apparent cell damage. Conversely, MPs are not internalized, and the effects on hASCs have to be ascribed to the release of ions in the culture medium, or to the reduced oxygen and nutrient exchange efficiency due to the presence of MP agglomerating around the cells. Notwithstanding the results depicting a heterogeneous scene that does not allow drawing a general conclusion, this work reiterates the importance of comparative investigations on MPs, NPs, and corresponding ions, and the need to continue the thorough verification of NP and MP innocuousness to ensure unaffected stem cell physiology and differentiation.

## 1. Introduction

It is well known that many human tissues, including skin, liver, muscle, pancreas, lung, adipose tissue, placenta, bone marrow, peripheral blood, as well as neural tissue, contain an undifferentiated cell population facilitating tissue repair and remodelling over the course of a lifetime [1,2]. The research on adult stem cells (SCs) began in the 1950s when researchers discovered that the bone marrow contained at least two kinds of SCs: hematopoietic and mesenchymal stem cells. In recent years, there has been a considerable increase in the comprehension of SC biology for their involvement in maintaining the homeostasis of healthy tissues or in tumour formation and proliferation. Furthermore, SCs have shown great potential in many medical applications, such as regenerative medicine, bone marrow transplantation, orthopaedic injuries, autoimmune diseases, and cardiovascular and liver diseases [3,4,5]. It is, therefore, evident how dangerous it could be to perturb SC homeostasis acting on their turnover, which may lead to SC depletion that, in turn, is responsible for pathological consequences [6]. In this scenario, a circumstance that has also to be taken into account is the exponential growth that nanotechnology has had in this last decade in several areas, including medical biotechnology within which the theranostics plays an important role. Theranostics is a discipline that combines diagnostic and therapeutic properties in the same compound [7]; this concept refers to the optimization of medical nanotechnologies, which are in search of the ideal system, able to recognize and target, in a peculiar way, the area of interest to promote an efficient site-directed action and provide accurate imaging for diagnostic follow-up. In this context, magnetic nanoparticles (NPs) with intrinsic theranostic characteristics can give an optimal solution [8,9,10,11]. Considering this overview, it is easy to imagine that the human body is easily exposed to the possibility of coming into contact, intentionally or not, with NPs that may enter the body, encounter SCs of different tissues, then act on them perturbing their physiology. Consequently, SCs might represent a possible weak point, and their susceptibility to NPs should be carefully evaluated. Moreover, appropriate assessment of the biological effects of NPs is particularly arduous as their in vitro and in vivo behaviours depend on several factors, including composition, shape, size, dosage, and route of exposure [12,13,14]. Although Ni represents a well-known problem for allergy and dermatitis [15], Fe, Co, and Ni are very promising for nanotechnological applications. In nanomedicine, for instance, iron and cobalt in particular, find applications as highly-sensitive contrast agents in magnetic resonance imaging (as an alternative to radionuclides), vectors for drug delivery, and theranostics, combining imaging analysis and hyperthermia therapy [8,10,16,17,18]. Moreover, we think that SCs, due to their higher sensitivity to the xenobiotic compounds, will play an increasingly important role in in vitro cell based tests for developmental toxicology studies, as well as target organ toxicity [6].

For all these reasons, and with the purpose to face some of these aspects, we have planned to study the behaviour of microparticles (MPs) and NPs of iron (Fe), cobalt (Co), and nickel (Ni), three transition metals of the group 8 of the periodic table, on SCs. In this paper, we reported our findings on cytotoxicity, morphology, cellular uptake, and gene expression of human adipose-derived mesenchymal stem cells (hASCs) exposed to zero-valent metal MPs, NPs, or salts.

## 2. Results

### 2.1. Micro- and Nanoparticle Characterization

**Figure 1:**
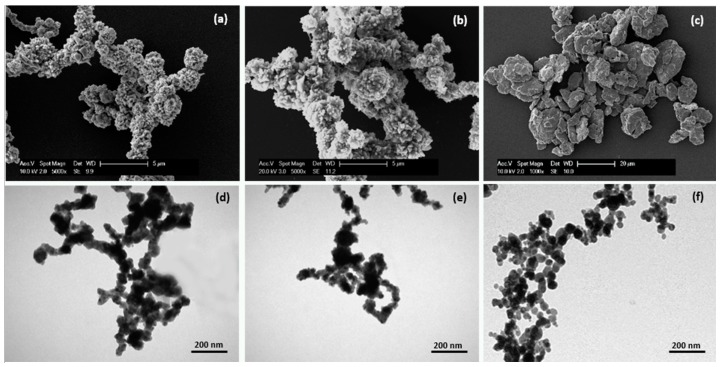
Scanning electron microscopy images of metal microparticles deposited on a glass coverslip: Fe (**a**), Co (**b**), and Ni (**c**) and transmission electron microscopy images of metal nanoparticles deposited on formvar carbon-coated grids: Fe (**d**), Co (**e**), and Ni (**f**). Certain heterogeneity in the size and shape is observed for all the particles.

The MPs and NPs used in this research were analysed by microscopy (Figure 1). Scanning electron microscopy images (SEM) observations displayed MPs with different sizes, irregular round shapes, and a strong tendency to cluster in aggregates. The MPs showed an average diameter of 6 µm ± 1.5; 0.8 µm ± 0.15 and 4 µm ± 0.5 for Fe, Co, and Ni, respectively, confirming the label information (Figure 1a–c). Transmission electron microscopy (TEM) analysis of NPs showed particles of different shape whose size is between 10 and 50 nm (Figure 1d–f). 

### 2.2. Cell Viability

**Figure 2:**
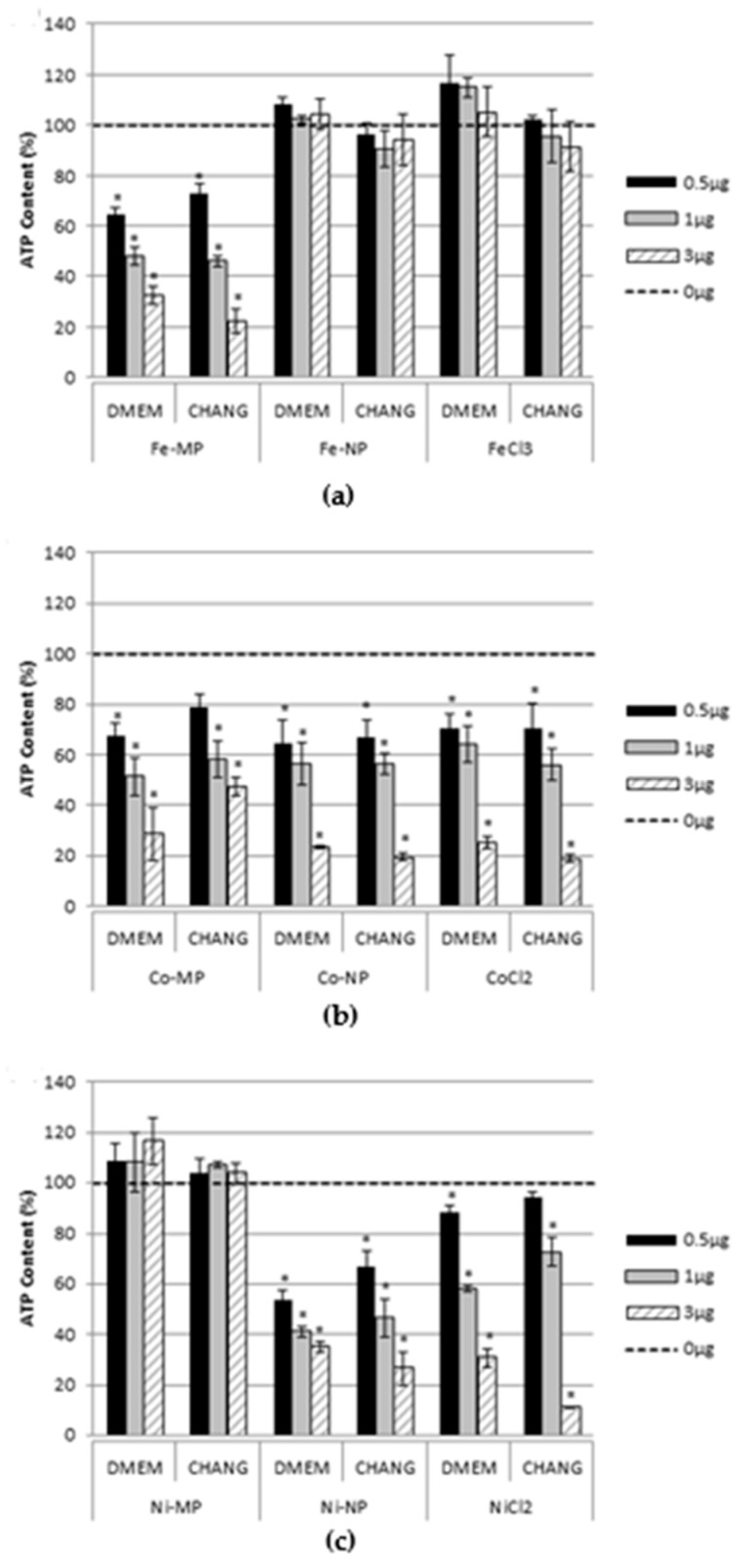
Percentage of ATP content, normalized to control, in hASCs exposed to Fe, (**a**), Co (**b**), and Ni (**c**), microparticles (MPs), nanoparticles (NPs), or salt for 96 h. Bars represent standard errors. * Dunnett’s test, *p* < 0.05. Each plotted value is the mean of three independent experiments.

Figure 2 indicates cytotoxicity induced by Fe, Co, and Ni MPs, NPs, and ions evaluated measuring cellular adenosine triphosphate (ATP) content after 96 h exposure of hASCs maintained in CHANG MEDIUM^®^ C or Dulbecco’s Modified Eagle Medium/Dulbecco’s Modified Eagle Medium Nutrient Mixture F12 (DMEM/DMEM-F12) to be sure that the cell toxicity was due only to the MPs, NPs, and ions exposure and not influenced by the cell culture medium. The response was, in most cases, concentration-dependent, and no differences were evidenced between the two culture media. As expected, the effects were significantly different among the three metals. The most striking data are those referred to Fe and Ni MPs (Figure 2a,c) which evidenced a high toxicity for Fe MPs and no effect for Ni MPs compared to the other two formulations. Conversely, Co MPs, NPs, and CoCl_2_ elicited a dose-dependent response similar in all the three formulations (Figure 2b). In addition, NiNPs exposure showed a cell toxicity comparable to that of CoNPs.

### 2.3. Cellular Uptake and Morphology

**Figure 3:**
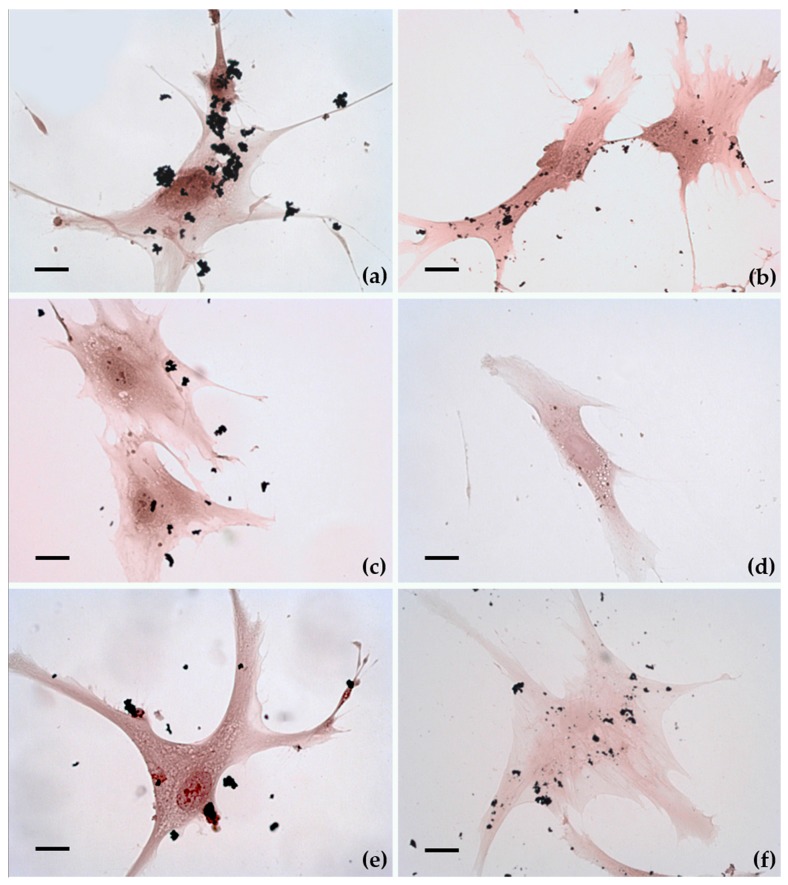
Optical microscopy photographs of hASCs exposed for 24 h to micro and nanoparticles and stained with haematoxylin-eosin solution. Micro- and nanoparticles are visible as black corpuscles. FeMPs (**a**), FeNPs (**b**), CoMPs (**c**), CoNPs (**d**), NiMPs (**e**), and NiNPs (**f**). All cells maintain the classical fibroblast-like morphology. Original magnification: 20×. Bar indicates 40 µm.

Figure 3 shows the cellular uptake of MPs and NPs evaluated by optical microscopy after 24 h of exposure. Although Fe, Co, and Ni MPs appear different in shape and size, none of them appeared to enter the cells (Figure 3a,c,e). Conversely, several Fe, Co, and Ni NPs seemed to be inside the cells (Figure 3b,d,f). Cell appears to maintain the classical fibroblast-like morphology characteristic of unexposed hASCs [4].

**Figure 4:**
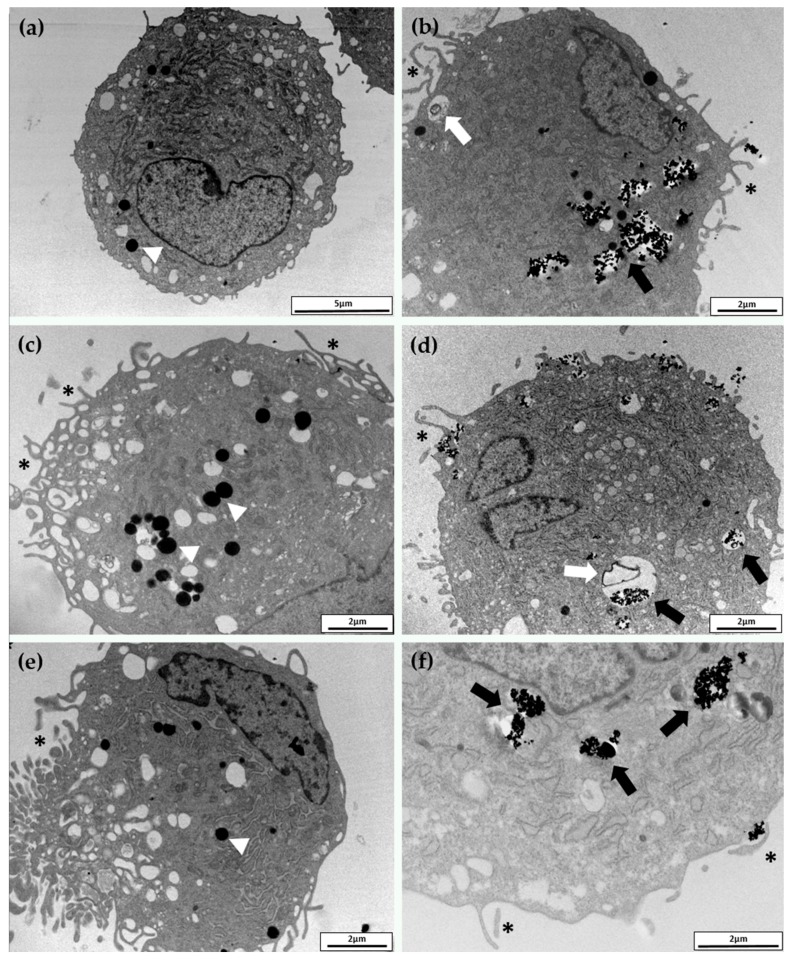
Transmission electron microscopy images of typical hASCs exposed for 24 h to FeMPs (**a**), FeNPs (**b**), CoMPs (**c**), CoNPs (**d**), NiMPs (**e**), and NiNPs (**f**). NPs were identified as high-electron-density objects when inside the cell and localized inside the vesicles (black arrows). No differences were observed among the three considered NPs and the internalization appeared to be nonspecific. White arrows indicate lysosomes of different sizes containing amorphous material. Several pronounced pseudopodia-like protrusions (*) are present both in NP- and in MP-exposed cells. Nuclei and mitochondria do not contain NPs. Arrowheads indicate lipid droplets.

TEM analysis, reported in Figure 4, confirmed the optical microscopy observations. Both MPs and NPs, at the concentration and exposure time used in these experiments, provoked some ultrastructural modifications, but no severe cellular damage. The cell body was rounded and the nucleus was large, indented, and eccentric, with abundant euchromatin and a prominent nucleolus (not shown in these pictures). Even though we have not observed NPs inside the nucleus, nor in the mitochondria, we cannot exclude their internalization in these compartments. Ultrastructural analysis seemed to also exclude rough endoplasmic reticulum (RER) and Golgi apparatus involvement compared to unexposed cells. Vesicles and lysosomes of different size, containing low electron-dense material, were present in the cytoplasm (Figure 3b,d). These characteristics suggested that exposed hASCs maintained the typical characteristics [19]. However, it is clear that NPs were massively internalized as agglomerations, mainly by endocytic mechanisms (Figure 4b,d,f). Once internalized, most of the NPs remained inside the cytoplasmic vesicles which may also contain amorphous cellular material (Figure 4b,d). No differences were observed among the three considered NPs and the internalization appeared to be nonspecific. In these pictures, NPs are identified as high electron density material as NPs preserve the morphology observed in the cell-free environment (Figure 1). The cell membrane is characterized by pronounced pseudopodia-like protrusions, especially in MPs exposed cells that also show several empty vacuoles (Figure 4a–c). These findings represent the typical alterations of an early apoptotic state. 

**Figure 5:**
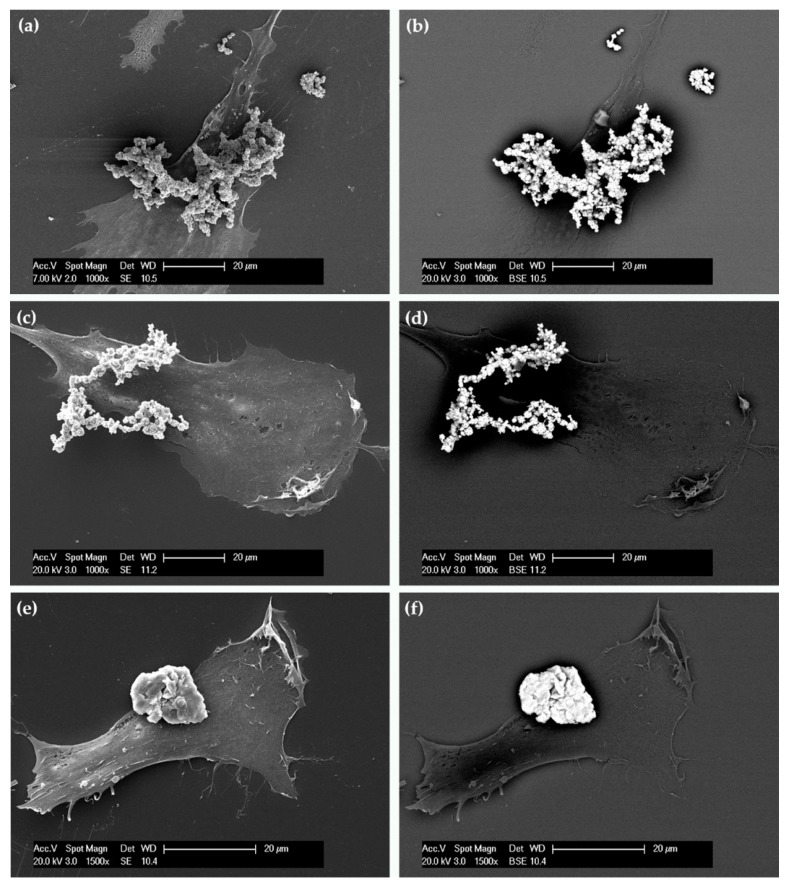
Scanning electron micrographs of typical hASCs exposed for 24 h to FeMPs (**a**,**b**), CoMPs (**c**,**d**), and NiMPs (**e**,**f**). The same field of view is shown with secondary electron (SE, left) and backscattered electron (BSE, right) imaging. In BSE imaging, the clusters of metal microparticles appear as bright spots.

Conversely, these cells seemed unable to internalize the MPs (Figure 4a,c,e). TEM observations were supported by SEM analysis. In particular, important information was obtained by using secondary electrons (SE) (Figure 5a,c,e) and backscattered electrons (BSE) (Figure 5b,d,f). Figure 5b,d,f show micrographs of cells covered with large electron-dense materials (bright spots) confirming that all the MPs resided around and not inside the cells. 

### 2.4. Gene Expression

**Figure 6:**
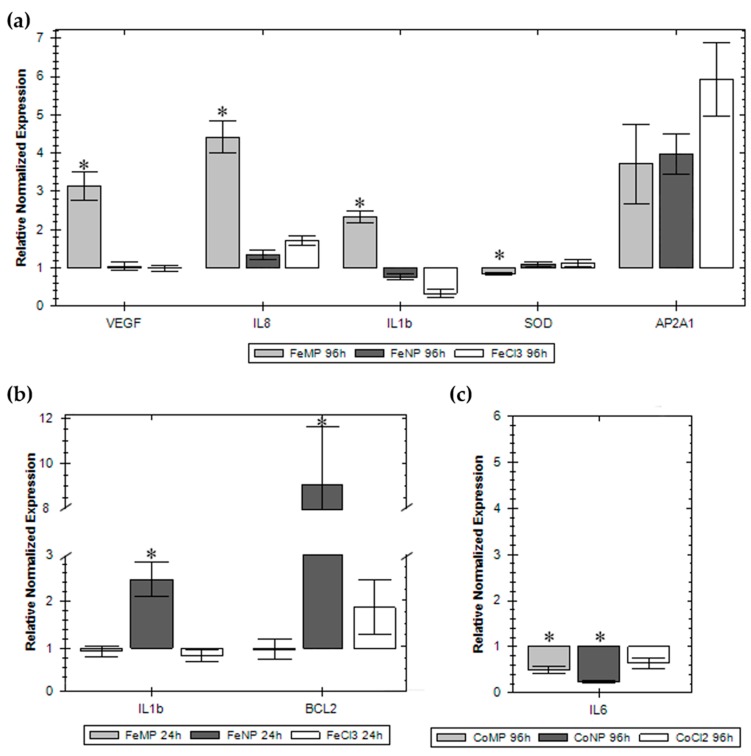
Real-time PCR of the most significant gene expression modifications**.** *VEGF*, *IL8*, *IL1b*, *SOD* and *AP2A1* in cells exposed for 96h to Fe MPs, NPs and ion (**a**). *IL1b* and *BCL2* in cells exposed for 24 h to Fe MPs, NPs and ion (**b**). *IL6* in cells exposed for 96h to Co MPs, NPs and ion (**c**). Normalization has been done with at least three housekeeping genes. The mRNA expression is reported as the fold change compared to the unexposed cells whose expression has been fixed at one. * Dunnett’s test, *p* < 0.05. Each plotted value is the mean of three independent samples.

The genes considered in this research are those implicated in some cell processes, such as oxidative stress, apoptosis, inflammatory response, neovascularization, tissue regeneration, and internalization. In Figure 6 we have reported only the results of those genes whose expression was significantly changed. As reported in Figure 6a, Fe MPs induced upregulation of vascular endothelial growth factor A (*VEGFA*), interleukin 8 (*IL8*), interleukin 1b (*IL1b*), and a downregulation of superoxide dismutase 1 (*SOD*) after 96 h of exposure. Although not statistically significant, iron, in all its formulation, induced the expression of adaptor-related protein complex 2 alpha 1 subunit (*AP2A1*). Figure 6b shows an upregulation of *IL1b* and B-cell lymphoma 2 (*BCL2*) genes after 24 h of Fe NP exposure. Surprisingly, cobalt caused only the downregulation of interleukin 6 (*IL6*) expression after 96 h of exposure (Figure 6c).

## 3. Discussion

Differences in the toxicological characteristic of nanosized and non-nanosized particles have been extensively studied for titanium dioxide particles [20,21]. Conversely, formulations, based on iron, cobalt, and nickel, which also have several applications that have not received the same attention. Furthermore, notwithstanding the increasing literature on nanotoxicity, still scarce and contradictory are the papers that deal with possible interferences of nanomaterials on SCs. Some authors did not find any influence of NPs on SC viability/proliferation and differentiation [22,23,24,25]; other researchers have instead observed marked effects [26,27,28]. In this paper, we have faced a comparative study based on size–effect of commercially zerovalent iron, cobalt, and nickel MPs and NPs using the hASCs as a model. There are several factors that intervene to maintain the SC microenvironment which are critical: oxygen tension, hormones, growth factors, cytokines, nutrients and extracellular matrix; this is why we first evaluated cell viability of hASCs maintained in two different culture media. Our results suggested that, in our experimental conditions, the medium did not influence the behaviour of the exposed hASCs. Even though our experiments confirmed the previous results on cell viability for the NP formulation whose cytotoxicity ranking was CoNPs > NiNPs > FeNPs, conversely, hASCs appeared more sensitive to NP exposure. As cytotoxicity could be ascribed to metal dissolution in growth media during exposure, cobalt, which easily releases ions when placed in aqueous systems, displayed the same effect for the three formulations [14]. Unforeseen was the high cytotoxicity shown by FeMPs, and this behaviour was supported by our results on gene expression but, to our knowledge, not from the literature [29,30]. The NiMPs, often used as electrodes catalysts or components of catalytic converters, resulted in a lack of toxicity [31,32]. The Ni ion is introduced with the diet and it is a component of some enzymes of our body; as in the culture medium, NiNPs release less than 10% as Ni^2+^ [14], a low toxicity would be expected . On the contrary, as also reported by Ahamed [33], our results showed that both NiNPs and Ni ions exert significant effects on hASCs. To note, the medium does not influence cytotoxicity.

Our investigations, conducted by TEM, confirm that zero-valent metal NPs are mainly internalized by endocytosis and persist in the cytoplasm inside vesicles [34,35]. These results are also supported by our previous paper in which we have proved that cobalt oxide NPs, but not zero-valent cobalt NPs, can cross the plasma membrane [36]. Conversely, SEM analysis proved that MPs are not internalized; consequently, any effects on hASCs have to be ascribed to the release of ions in the culture medium, or to the reduced oxygen and nutrients exchange efficiency, due to the presence of MP agglomerate around the cells. As assessed with various in vitro assays, exposed cells can die by apoptosis and/or necrosis, phenomena that depend on the concentration, chemical nature, and size of metal NPs [37]. At low toxic doses, however, metal NPs can induce pro-inflammatory effects. The literature reports that macrophages exposed to AgNP were rapidly induced to secrete *IL8* and oxidative stress genes, such as hemeoxygenase-1 and heat shock protein 70 (*Hsp70*), in a size-dependent way [38]. Moreover, the ability to induce the innate immunity, measured as production of *IL1b*, and the induction of inflammasomes was higher for small AgNPs and ZiONPs [39]. 

The gene expression of the exposed hASCs conducted in this research does not appear heavily influenced nor strictly related to the cytotoxicity dose-response curves or to the size, as well as to the nature, of the compounds used in this study. The overall picture shows modifications in mRNA expression of some genes involved in the inflammatory response; the main noteworthy changes are due to FeMPs after 96 h of exposure that clearly induce *IL8*, *IL1b*, and *VEGFA*. Even though not statistically significant and independently to the size, iron raises the mRNA expression of *AP2A1*, a protein involved in clathrin-dependent endocytosis; consequently, its induction may be related to an increase in NP endocytosis [40]. CoMPs and CoNPs, after 96 h of exposure, influenced *IL6*, a multifunctional cytokine [41] that, together with *VEGFA*, it is known to be a putative paracrine factor secreted by hASCs and involved in cytoprotection, proliferation, and angiogenesis [42]. None of the formulations used affected stress-related genes (catalase, *CAT*; *SOD*, metallothionein 1A, *MT1A*; *Hsp70*) even though their main function is to protect cells from various injuries, such as elevated temperature, mechanical damage, hypoxia, and reactive oxygen species [14]. Lastly, the genes related to apoptosis (tumour protein 53, *P53*; caspase 3, *CASP3*; early growth response protein 1, *EGR1*; *BCL2*) are not influenced by the treatment; the only exception was the increase of BCL2, a protein that blocks the programmed cell death and apoptosis pathway, after 24 h exposure to FeNPs. 

It is known that each cell line has its own sensitivity to NP and MP exposure and, notwithstanding the multidisciplinary approach adopted in this research, the results depict a heterogeneous scene with regard to the interactions of nanoparticles, microparticles, and salts with hASCs. In this context, is difficult to depict a general conclusion. Nevertheless, it is rather clear that parameters such as chemistry, shape, and size sometimes greatly affect the behaviour of hASCs, but do not alter their natural differentiation time. Probably, MPs lead cells to an apoptotic state due more to the presence of agglomerates around the cells, than to their composition. This work reiterates the importance of comparative investigations on MPs, NPs, and the respective ions to avoid experimental artefacts in the evaluation of nanotoxicity. Furthermore, we think that it is necessary to stress the adoption of in silico toxicology methods to conceive informative models on the in vitro and in vivo effects. Therefore, it will be indispensable to continue the thorough control of NPs’ and MPs’ innocuousness to ensure that they do not perturb SC homeostasis acting on their physiology, differentiation, and turnover that may lead to SC depletion that, in turn, is responsible for pathological consequences.

## 4. Materials and Methods

All subjects gave their informed consent for inclusion before they participated in the study. The study was conducted in accordance with the Declaration of Helsinki, and the protocol was approved by the Ethics Committee of University of Insubria, Varese.

### 4.1. Micro- and Nanoparticle Characterizations

MPs were purchased from American Elements (Los Angeles, CA, USA) with the following characteristics: FeMPs (purity 99.9%, aerodynamic particle size < 10 µm), CoMPs (purity 99.8%, aerodynamic particle size < 2 µm), and NiMPs (purity 99.9%, aerodynamic particle size < 5 µm). NPs were purchased from IOLITEC (Heilbronn, Germany) with the following characteristics: FeNPs (purity 99.9%, aerodynamic particle size < 25 nm), CoNPs (purity 99.8%, aerodynamic particle size < 28 nm) and NiNPs (purity 99.8%, aerodynamic particle size < 20 nm). 

For MP characterization, 5 μL of a diluted ethanol suspension were deposited on a glass coverslip and analysed by SEM (FEI XL-30 FEG, Eindhoven, The Netherlands) operated at an acceleration voltage of 7 kV. Conversely, 5 μL of a diluted ethanol suspension of NPs were deposited on a formvar carbon-coated copper grid and observed by TEM microscope (Morgagni, Philips, Eindhoven, The Netherlands) operating at 80 kV [14].

### 4.2. Patient Samples

hASCs were isolated from mammary adipose tissue, obtained from five healthy women (average age 43 ± 4) who underwent surgery for gigantomasty at the “Ospedale di Circolo”, Varese, Italy. All patients were in good health who have not undergone to heavy weight loss diet, their body mass index (BMI) was from 18.8 to 29 kg/m^2^ (mean 24.58 ± 5.24 kg/m^2^), non-smokers, without a history of metabolic disorders, and not receiving medications at the time of surgery. Gigantomastia allowed us to obtain large amounts of adipose tissue from the same depot. Furthermore, the severe selection of the patients guaranteed adipose tissue of good quality and rich in hASCs. 

### 4.3. hASC Isolation and Culture

The stromal cellular fraction of human mammary adipose tissue, obtained according to Gronthos and Zannettino protocol [43], was divided in two fractions. Half of these cells were seeded in a flask with complete 1:1 DMEM/DMEM-F12 medium (Sigma Aldrich, Milan, Italy) containing 10% FBS, 2 mM L-glutamine, 1% penicillin/streptomycin, and 0.1% gentamicin. The remaining fraction of cells was seeded in CHANG C medium (Irvine Scientific, Santa Ana, CA, USA) supplemented with 2 mM L-glutamine, 1% penicillin/streptomycin. After 6 h non-attached cells were removed and adherent stem cells were fed with fresh medium. hASCs were characterized by flow cytometry, as reported in our previous paper [4]. For all subsequent experiments hASCs were used at passage 5.

### 4.4. Cell Exposure and Viability

Cell viability was determined measuring ATP content by the CellTiter-Glo^®^ Luminescent Cell Viability Assay (Promega, Milan, Italy) according to the manufacturer’s instructions and as reported by Bava et al. [44]. This experiment was conducted in hASCs cultured in DMEM/DMEM-F12 and CHANG medium. Briefly, 300 cells were seeded into 96-well plates and treated after 24 h. Cells were exposed for 96 h in increasing concentrations (0, 0.5, 1, 3 μg/200 μL) of Fe, Co, or Ni MPs and NPs and their corresponding salts (FeCl_3_, CoCl_2_·6H_2_O, NiCl_2_). MPs and NPs were resuspended in fresh culture media and then dispersed by an ultrasonic bath for 15 min before each treatment. The range of concentrations of each metal has been determined in preliminary experiments. After the exposure, plates were equilibrated for 30 min at room temperature then the culture medium was replaced by 1:1 phosphate buffered saline (PBS):CellTiter-Glo reagent. Plates were shaken for 2 min and left at room temperature for 10 min before recording luminescent signals using the Infinite F200 plate reader (Tecan Group, Männedorf, Switzerland). 

### 4.5. Uptake and Morphology

These experiments were performed in steady state. For optical microscopy 5000 cells were cultured in DMEM on 24 mm^2^ coverslip into a six-well culture plates, cultivated at 37 °C in 5% CO_2_ and then exposed to 1.2 μg/200 μL of Fe, Co, or Ni MPs and NPs for 24 h. After the exposure, MPs and NPs were removed; cells were washed with PBS, fixed for 10 min with methanol at room temperature, and then stained with haematoxylin-eosin solution according to the standard procedures.

For TEM analysis, 100,000 cells were cultured in a T25 flask, and exposed as above reported. After the exposure, MPs and NPs were removed; cells were washed with PBS, harvested, fixed, embedded as previously described [45], and finally observed by a Morgagni electron microscope (Philips, Eindhoven, The Netherlands) operating at 80 kV. For SEM observation, 1000 cells were cultured on a 24 mm^2^ coverslip, placed into a six-well culture plates, and treated as above. After the exposure, MPs and NPs were removed; cells were washed with PBS and fixed in Karnovsky solution (4% formaldehyde, 2% glutaraldehyde, 0.1 M sodium cacodylate, pH 7.2) for 1 h. Samples were then dehydrated with a series of ethanol (20%, 50%, 70%, 90%, 100%), treated with hexamethyldisilazane (HMDS), mounted on standard SEM stubs with conductive carbon-based adhesive, and gold-coated in an Emitech K-550 sputter-coater (Emitech Ltd., Ashford, UK) in a controlled argon atmosphere at a pressure of 0.1 mbar. All observations were carried out on a FEI XL-30 FEG field emission scanning electron microscope (FEI, Eindhoven, The Netherlands) operated at an acceleration voltage of 7 kV.

### 4.6. RNA Isolation, Reverse Transcription, and Real-Time PCR

A suitable number of cells (20,000–80,000) were seeded into six-well plates and exposed for 24 or 96 h to Fe, Co, or Ni MPs and NPs (exposure concentration was 1.2 μg/200 μL at 24 h and 0.6/200 μL at 96 h, referred to a cell ATP reduction of about 60–80%). Exposed and not exposed cells were then washed twice with PBS and harvested. We have also arranged a set of samples in which cells were exposed to FeCl_3_, CoCl_2_·6H_2_O, or NiCl_2_ at a nominal concentration of 6 and 3 μg mL^−1^ in terms of Fe, Co, and Ni. Total RNA was isolated using the Direct-zol RNA Miniprep (Zymo Research, Milan, Italy) according to the protocol, quantified by the QuantiFluor^®^ RNA System (Promega, Milan, Italy) and reverse transcribed by iScriptTM cDNA Synthesis Kit (Bio-Rad, Segrate, Italy) according to the manufacturer’s instructions. The Quantitative Real-Time PCR (qPCR) was performed with iTaq™ Universal SYBR^®^ Green Supermix (Bio-Rad, Segrate, Italy) technology using CFX Connect^®^ Real-Time PCR Detection System apparatus (Bio-Rad, Segrate, Italy). Specific primers were designed by Beacon Designer 7^®^ (Bio-Rad, Segrate, Italy) within their own sequence, and on an exon-exon junction (possibly separated by an intron of at least 1000 bp) in order to prevent genomic DNA amplification. The analysis was performed on genes involved in oxidative stress (*CAT*; *SOD*; *MT1A*; *Hsp70*), apoptosis (*P53*; *CASP3*; *BCL2*; *EGR1*), inflammatory response (*IL6*; *IL8*; *IL1b*), neovascularization, tissue regeneration (*HIF1α*; *VEGFA*), and internalization (*AP2A1*), see Table 1 [46,47,48,49,50,51,52]. Housekeeping genes (*ACTβ: β-actin*; *B2M: beta-2-microglobulin*; *GAPDH: glyceraldehyde-3-phosphate dehydrogenase*; *RPL13A: ribosomal protein L13A*; *RPS18: ribosomal protein S18*; *PPIA: peptidylprolyl isomerase A*) were selected as described in Palombella et al. [53]. qPCR reaction was set up with 300 nM of each primer and 5 ng of cDNA in a total volume of 10 µL. The thermocycler program included an initial hot start cycle at 95 °C for 3 min followed by 40 amplification cycles consisting of a denaturation step at 95 °C for 10 s and an annealing-extension phase at 60 °C for 30 s. A melt-curve was performed at the end of each run. Technical triplicates were run for all samples. 

### 4.7. Statistical Analysis

Cytotoxicity results were expressed as mean F ± SE. With respect to qPCR, the comparative ΔΔCt method was used to present normalized gene expression. All data were analysed with one-way Analysis of Variance ANOVA (α = 0.05), completed with Dunnett’s test (*p* < 0.05) in order to determine which groups (NPs, MPs, ions) were significantly different from control. Statistical significant differences was fixed at *p* ≤ 0.05 (*). Each plotted value is the mean of three independent experiments. 

## Figures and Tables

**Table 1:** Primers used in this work. Fw: forward, Rv: reverse.

Gene Name	Sequence 5′–3′		Amplicon Leght (bp)	NCBI Accession Number
*ACTβ*	Fw	ATGGGTCAGAAGGATTCC	78	NM_001101.3
	Rv	CTCGATGGGGTACTTCAG		
*B2M*	Fw	CTATCCAGCGTACTCCAA	93	NM_004048.2
	Rv	GAAACCCAGACACATAGC		
*GAPDH*	Fw	TTTGGCTACAGCAACAGG	107	NM_001289746.1
	Rv	GGTCTCTCTCTTCCTCTTG		
*RPL13A*	Fw	TATGAGTGAAAGGGAGCC	82	NM_001270491.1
	Rv	ATGACCAGGTGGAAAGTC		
*RPS18*	Fw	GAGGTGGAACGTGTGATC	109	NM_022551.2
	Rv	GGACCTGGCTGTATTTTC		
*PPIA*	Fw	AACCACCAGATCATTCCTT	86	NM_001300981.1
	Rv	GCGAGAGCACAAAGATTC		
*MT1A*	Fw	CTCCTGCAAGAAGAGCTG	87	NM_005946.2
	Rv	TTCTCTGATGCCCCTTTG		
*Hsp70*	Fw	AGGCGGAGAAGTACAAAG	85	NM_005345.5
	Rv	ATGTTGAAGGCGTAGGAC		
*CAT*	Fw	TACCCTCTCATCCCAGTT	85	NM_001752.3
	Rv	GGTCGAAGGCTATCTGTT		
*SOD*	Fw	CAGATGACTTGGGCAAAG	82	NM_000454.4
	Rv	CCAATTACACCACAAGCC		
*P53*	Fw	CCACCATCCACTACAACT	92	NM_000546.5
	Rv	GGAGTCTTCCAGTGTGAT		
*CASP3*	Fw	GAGGCCGACTTCTTGTAT	92	NM_004346.3
	Rv	CAAAGCGACTGGATGAAC		
*BCL2*	Fw	CCTTCTTTGAGTTCGGTG	98	NM_000633.2
	Rv	CAGGTACTCAGTCATCCA		
*EGR1*	Fw	GCAGAAGGACAAGAAAGC	94	NM_001964.2
	Rv	CGGGTAAGAGGTAGCAAC		
*HIF1α*	Fw	CAAGTCCTCAAAGCACAG	75	NM_001530.3
	Rv	TGGTAGTGGTGGCATTAG		
*VEGF*	Fw	GGAGTCCAACATCACCAT	80	NM_001171623.1
	Rv	GCTGTAGGAAGCTCATCT		
*IL6*	Fw	ACTCACCTCTTCAGAACG	113	NM_000600.3
	Rv	CCTCTTTGCTGCTTTCAC		
*IL8*	Fw	GCCAAGGAGTGCTAAAGA	103	NM_000584.3
	Rv	TGGTCCACTCTCAATCAC		
*IL1b*	Fw	CTACGAATCTCCGACCAC	90	NM_000576.2
	Rv	AACCAGCATCTTCCTCAG		
*AP2A1*	Fw	CTGGTGGAATGTCTGGAG	117	NM_014203.2
	Rv	GATGATGAGGCTGATGGT		
